# Electron cyclotron motion excited surface plasmon and radiation with orbital angular momentum on a semiconductor thin film

**DOI:** 10.1038/s41598-020-73725-6

**Published:** 2020-10-07

**Authors:** Yung-Chiang Lan, Chia-Hui Shen, Chih-Min Chen

**Affiliations:** Department of Photonics and Advanced Optoelectronic Technology Center, National Cheng Kung University, Tainan, 701 Taiwan

**Keywords:** Optics and photonics, Nanophotonics and plasmonics, Terahertz optics

## Abstract

In this work, surface plasmons (SPs) on a germanium (Ge) thin film in terahertz (THz) region that are excited by electron cyclotron motion (ECM) and the subsequent SP emission (SPE) by adding Ge gratings on the film are explored by finite-difference time-domain (FDTD) and particle-in-cell FDTD (PIC-FDTD) simulations. The optical properties of ECM-excited SPs are the same as those of SPs that are excited by electron straight motion (ESM). For operating at the flat band of SPs’ dispersion curve on the Ge film, changing the electron energy will only change the wavevector of SPs and hence the number of periods of SPs on the circular orbital. When the periodic gratings are deposited on the Ge film along the circular orbital of electrons, the emitted SPE contains the orbital angular momentum (OAM). The number of arms and chirality of the spiral patterns in phase map (i.e. the quantum number of OAM) of SPE are determined by the difference between the number of SPs’ periods and the number of gratings. Manipulations of the quantum number of OAM by changing the number of gratings for a fixed electron energy and by changing the electron energy for a fixed number of gratings are also demonstrated. This work provides an active OAM source and it is not required to launch circularly polarized beams or pumping beams into the structure.

## Introduction

Light beam can carry spin angular momentum (SAM) which is manifested in the left- and right-circularly polarized light. Light can also carry orbital angular momentum (OAM) which displays a helical phase front in its Poynting vector (i.e. the Poynting vector has the form of $\exp ( - i\,\ell \varphi )$, where $\varphi$ is the azimuthal coordinate in the beam’s cross section and ℓ can take any integer value)^1–4^. The OAM beam has ℓ intertwined helical phase fronts and when it is made to interference with a plan wave, it will produce a spiral intensity patter with ℓ arms (The integer ℓ is also called the quantum number of OAM^1,5,6^). There are three methods to generate light beams with OAM. The first and most original method is to convert a Hermite–Gaussian beam into a Laguerre-Gaussian beam by using cylindrical lenses^1,7^. The second method is based on the SAM-OAM coupling for a circularly polarized beam incident into the structure with a phase delay in the $\varphi$ direction. The phase-delayed structure can be a dislocation hologram (forked diffraction gratings)^2,4,8^, a spiral phase plate^4,9,10^, a liquid crystal/polymer Q-plate^11–13^, or a metasurface^14,15^. The third method is to use a ring resonator which supports the whisper gallery modes (WGMs)^16,17^. When adding periodic gratings on the top of the resonator, the emission wave carries OAM. However, all these OAM sources require that circularly polarized beams or pumping beams are incident into the structures and then converted into the beams with OAM. In the above mentioned methods, the forked diffraction gratings and metasurface structures make use of the non-uniform gratings. The ring resonators which supporting WGMs with gratings employ the uniform (periodic) gratings. The advantage of non-uniform gratings is that the phase change of the emitted radiation can be controlled more precisely. However, it is easier for designing uniform gratings.

Surface plasmons (SPs) are the electromagnetic waves that propagate at the plasmon-dielectric interface which are caused by free electrons in the plasmon to perform coherent oscillation^18^. SPs can be excited by light with using attenuated total reflection configuration or grating structure for matching the phase velocities of light and SPs. SPs can also be excited by a relativistic electron beam with a straight motion owing that the dispersion curves of electron beam and SPs intercept with each other^19–21^. Furthermore, the electron straight motion (ESM) excited SPs can be further transferred into light when they pass through the periodic gratings. A moving electron can perform the cyclotron motion when an external magnetic field is applied with its direction being perpendicular to that of the electron’s motion. Recently, using the electron cyclotron motion to generate the radiation with OAM in millimeter-wave regime in a cylindrical cavity^22^ and in X-ray regime in free space^23^ have also been proposed. The electron cyclotron motion (ECM) may also excite SPs on a plasmon structure. When adding the periodic gratings on the plasmon structure along the SPs’ orbital, radiation may be further extracted. Furthermore, because the electron possesses the OAM, the excited SPs and subsequent emission of radiation may also carry the OAM. However, these phenomena have never been investigated. Notably, SPs on a gold nanocylinder and on a cylindrical monolayer graphene with dielectric loading that are excited by a cyclotron electron beam (CEB) have been investigated^24,25^. In these structure, the SPs possess TM_m_ and HEM hybrid modes. When they are excited by CEB, they propagate along a spiral trajectory on the surface of the cylinder together with CEB. Furthermore, the SPs can transform into radiation directly. Conversely, for the planar plasmon structure, the only SPs modes are TM_0_ and TM_1_. Moreover, they cannot transform into radiation except for adding periodic gratings. Therefore, these two SPs are essentially different.

In this work, SPs on a germanium (Ge) thin film in THz region excited by ECM and subsequently transferred into radiation possessing OAM with adding Ge gratings on the film are investigated by finite-difference time-domain (FDTD)^26^ and particle-in-cell FDTD (PIC-FDTD)^27^ simulations. The optical properties of ECM-excited SPs are examined. Generations of radiation with OAMs are also observed and elucidated by inspecting the optical vortices formed in the phase distributed patterns of electric fields. Finally, manipulations of quantum number of emitted beam by changing the number of gratings with a fixed electron energy and by changing the electron energy with a fixed number of gratings are demonstrated.

## Results

**Figure 1:**
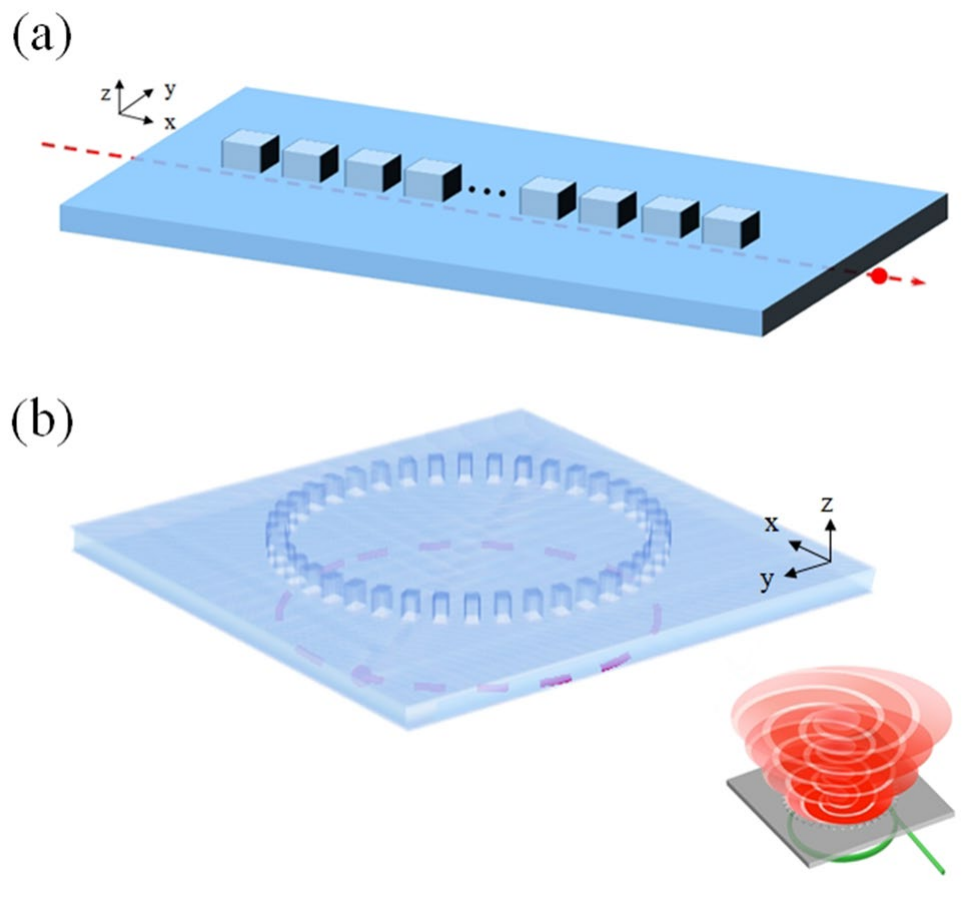
Schematic diagrams of simulation structures. (**a**) Simulated structure for an electron bunch moving along x-direction and under a Ge thin film. Periodic Ge gratings are deposited on the film also along x-direction. (**b**) Simulated structure for an electron bunch performing cyclotron motion under a Ge thin film with Ge gratings on the film along the electron’s circular orbital. Inset in (**b**): Schematic plot of emission of OAM under this configuration.

The schematic diagrams of simulation structures that are investigated in this work are presented in Fig. 1a and b. Figure 1a plots the simulated structure for an electron bunch moving along x-direction and under the Ge thin film. The periodic Ge gratings are deposited on the film also along x-direction. Figure 1b presents the simulated structure for an electron bunch performing the cyclotron motion under a Ge thin film with Ge gratings on the film along the electron’s circular orbital. The inset in Fig. 1b shows the schematic plot of emission of OAM under this configuration. The thicknesses of Ge film in Fig. 1a and b are both 6 μm. The length, width and height of the Ge gratings are 20 μm, 14 μm and 6 μm, respectively. The gyroradius ($r_{g}$) of the electron bunch in Fig. 1b is set as 200 μm. The total number of Ge gratings along the circular orbital, $q$, is changed between 35 and 45. The energy of electron bunch varies between 22 and 39 keV. However, to keep the gyroradius of electron bunch unchanged, the magnitude of applied z-directional magnetic field (Bz) is adjusted according to $r_{g} = \frac{m\,\upsilon }{{\left| e \right|\,B_{0} }}$, where $m$, $\upsilon$, $e$ and ${\text{B}}_{{0}}$ are the electron’s mass, the electron’s velocity, the electron’s charge, and the magnitude of applied magnetic field, respectively. Table 1 lists the values of electron energy, $\beta$ ($\beta \, = \,\upsilon /c$, *c* is speed of light), ${\text{B}}_{{0}}$, electron cyclotron frequency ($\omega_{c}$) and number of periods of SPs in the circular orbit ($p$, it will be explained later) that are used in the simulation. The simulation method and detailed settings are given in the section of “Methods and Materials”. The optical property of Ge is described by Drude model whose plasma and collision frequencies are also shown in “Methods and Materials”.

**Table 1:** The values of electron energy, $\beta$, applied external magnetic field (*B*_*0*_), electron cyclotron frequency (*ω*_c_) and number of periods of SPs in the circular orbit (*p*) that are used in the study.

Electron energy (keV)	β	*B*_*0*_ (Tesla)	*ω*_c_ (rad/s)	*p*
38.99	0.370	3.40	5.547 $\times$ 10^11^	35
36.62	0.360	3.29	5.393 $\times$ 10^11^	36
34.46	0.350	3.19	5.247 $\times$ 10^11^	37
32.50	0.341	3.09	5.109 $\times$ 10^11^	38
30.70	0.332	3.00	4.978 $\times$ 10^11^	39
29.06	0.324	2.92	4.854 $\times$ 10^11^	40
27.54	0.316	2.84	4.735 $\times$ 10^11^	41
26.14	0.308	2.77	4.623 $\times$ 10^11^	42
24.85	0.301	2.70	4.515 $\times$ 10^11^	43
23.66	0.294	2.63	4.413 $\times$ 10^11^	44
22.55	0.288	2.56	4.314 $\times$ 10^11^	45

**Figure 2:**
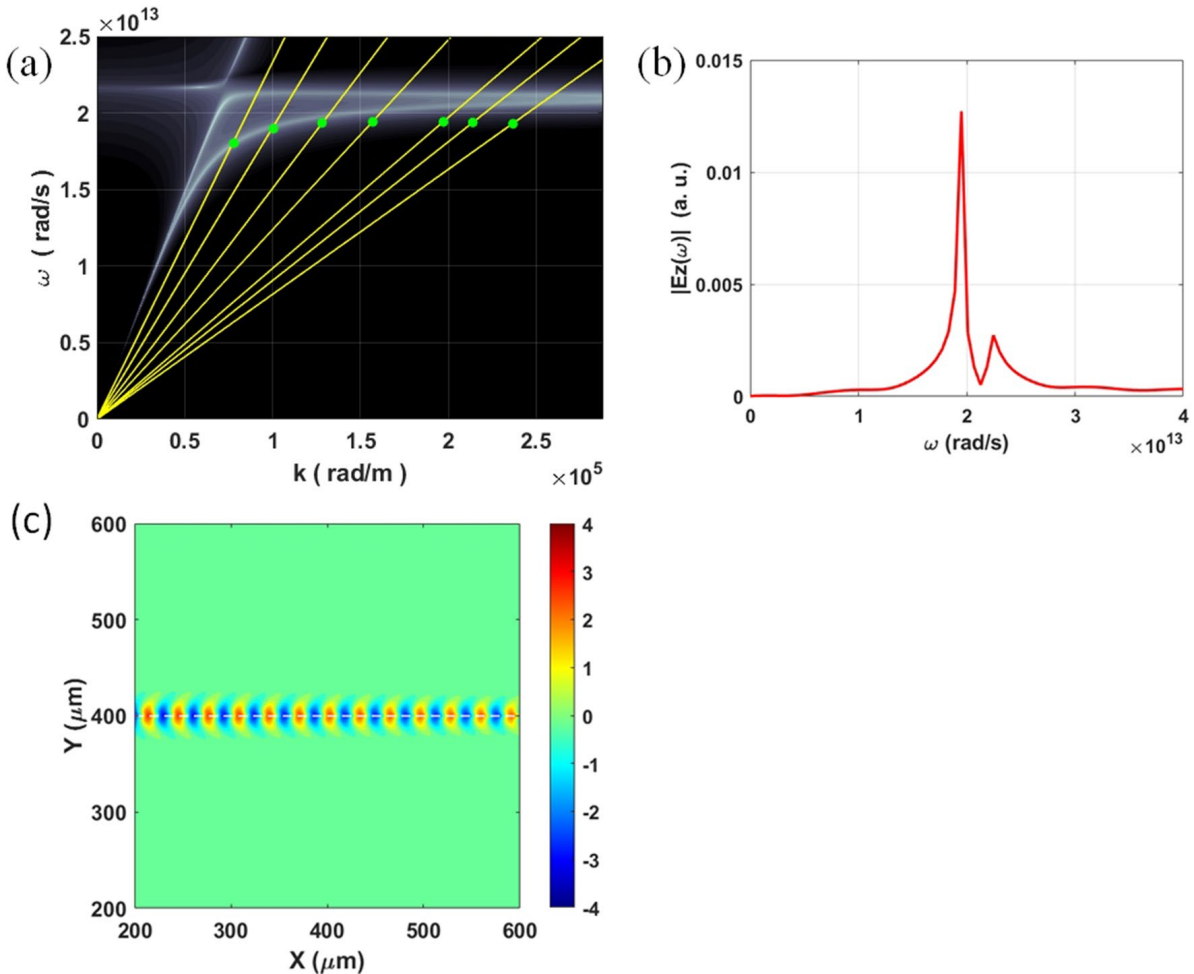
Simulation results for ESM-excited SPs on Ge film. (**a**) Calculated dispersion curves of SPs on a Ge thin film with the thickness of 6 μm (white lines) and electron beams with various energies (yellow lines). The green points denote FDTD simulated dispersion relations of SPs excited by the electron bunch. (**b**) Simulated frequency spectrum of Ez field of ESM-excited SPs with the electron energy of 29 keV (measured on the top surface of the Ge film). (**c**) Simulated contours of Fourier transform of ESM-excited Ez fields at 3 THz on the top surface of the Ge film.

The properties of SPs on the Ge thin film excited by an electron bunch with a straight trajectory under the film (see Fig. 1a) are examined first. The dispersion relation of SPs on a Ge film is expressed as^28^

1 $$\left( {\frac{{\varepsilon_{m} \left( \omega \right)}}{{\varepsilon_{a} }}\frac{{\alpha_{a} }}{{\varepsilon_{m} }} + 1} \right)^{2} = \left( {\frac{{\varepsilon_{m} \left( \omega \right)}}{{\varepsilon_{a} }}\frac{{\alpha_{a} }}{{\varepsilon_{m} }} - 1} \right)^{2} e^{{ - 2\alpha_{m} d}}$$

where $\varepsilon_{m} (\omega )$ and $\varepsilon_{a}$ denote the relative permittivities of Ge and air, respectively; $\alpha_{a} = \sqrt {k_{sp}^{2} - \varepsilon_{a} (\omega /c)^{2} }$, $\alpha_{m} = \sqrt {k_{sp}^{2} - \varepsilon_{m} (\omega )\,(\omega /c)^{2} }$ and $k_{sp}$ is the wavevector of SPs; *d* denotes the thickness of Ge film. Figure 2a plots the calculated dispersion curves of SPs on a Ge film with the thickness of 6 μm based on Eq. (1). In Fig. 2a, the yellow lines are the dispersion curves of an electron bunch with the energies from right to left to be 20 keV, 25 keV, 30 keV, 50 keV, 80 keV, 150 keV and 300 keV. The green points in Fig. 2a are the FDTD simulated dispersion relations of SPs excited by various electron energies. They agree with the calculated dispersion relations (i.e. the intercepting points of white and yellow lines in Fig. 2a). Figure 2a also shows that the dispersion curves are almost flat when the electron energy is smaller than 50 keV. It implies that, in the flat region, changing the electron energy will only change the value of $k_{sp}$. Figure 2b presents the simulated frequency spectrum of z-component electric field (Ez) of ESM-excited SPs with the energy of 29 keV. It shows that the peak frequency ($f_{sp}$) of the excited SPs (and the subsequent radiation when the period gratings are deposited on the film) is 3.0 THz ($1.9 \times 10^{13} \;rad/s$). Figure 2c plots the simulated contours of Fourier transform of Ez field at the frequency of 3 THz on the top surface of the Ge film. From Fig. 2c, the calculated $k_{sp}$ at 3 THz is $2.0 \times 10^{5}$ rad/m. Based on this value, there are total 40 periods of SPs on a 1257-μm-long (= $2\pi \times r_{g}$) Ge film.

**Figure 3:**
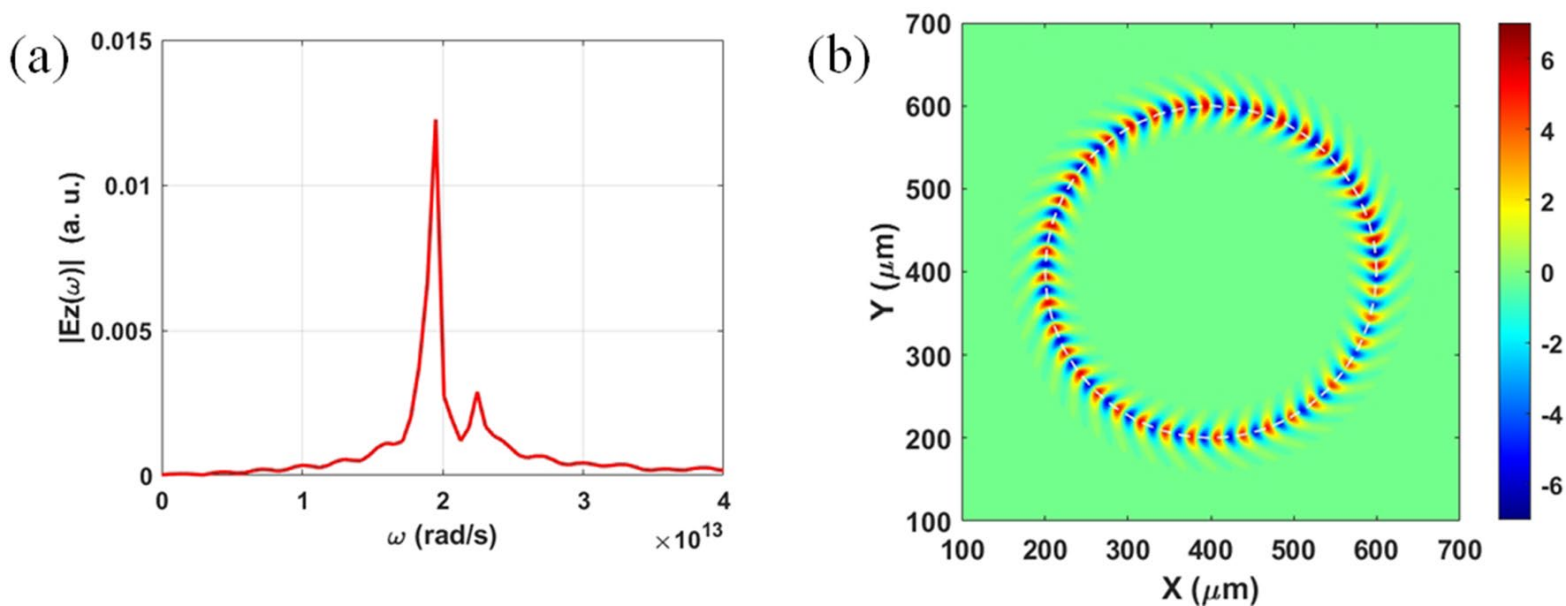
Simulation results for ECM-excited SPs on Ge film. (**a**) Simulated frequency spectrum of Ez field of ECM-excited SPs with the electron energy of 29 keV (measured on the top surface of the Ge film). (**b**) Simulated contours of Fourier transform of ECM-excited Ez fields at 3 THz on the top surface of the Ge film.

Next, the properties of ECM-excited SPs on a Ge film for an incident 29-keV electron bunch are investigated by FDTD and PIC-FDTD methods (see Fig. 1b and Table 1, $r_{g} = 200\;\mu m$). Figure 3a plots the simulated frequency spectrum of Ez field of SPs excited by ECM with the energy of 29 keV. The peak frequency of SPs is 3 THz which is the same as that of SPs excited by ESM. Figure 3b presents the simulated contours of Fourier transform of Ez field at 3 THz on the top surface of the Ge film. Figure 3b displays that there are total 40 periods of SPs along the electron’s circular orbit (i.e. $p = 40$ in Table 1). It reveals that $k_{sp}$ of ECM-excited SPs is also the same as that of ESM-excited SPs. This result can be ascribed to the following two reasons. First, for the planar Ge film, the only SPs modes are TM_0_ and TM_1_ [the dispersion relation is expressed in Eq. (1)]. The electron bunch mainly excites the lower-frequency TM_0_ mode. There are no other high-order SPs modes in this structure. Second, in our design, the electron cyclotron frequency is much smaller than the frequency of SPs and the circumference of ECM is much larger than SPs’ wavelength. It is also noted that ECM can emit radiation directly. However, the frequency of radiation of ECM is much smaller than that of SPs. Further, the radiation of ECM cannot excite SPs directly because of large wavevector mismatch between them. Therefore, the radiation from ECM has little effect on the excited SPs. Besides, the PIC-FDTD simulation results also show that the electron energy is reduced by around 0.007% during one cycle. Therefore, $f_{sp}$ and $k_{sp}$ of ECM-excited SPs are unchanged in a few cycles. The movie of electron bunch to perform cyclotron motion under a Ge film and its excitation of SPs on the film (by observing the Ez field on top surface) from PIC-FDTD simulation (29-keV electron energy and $r_{g} = 200$ μm) can be found in the Supplementary Information.

**Figure 4:**
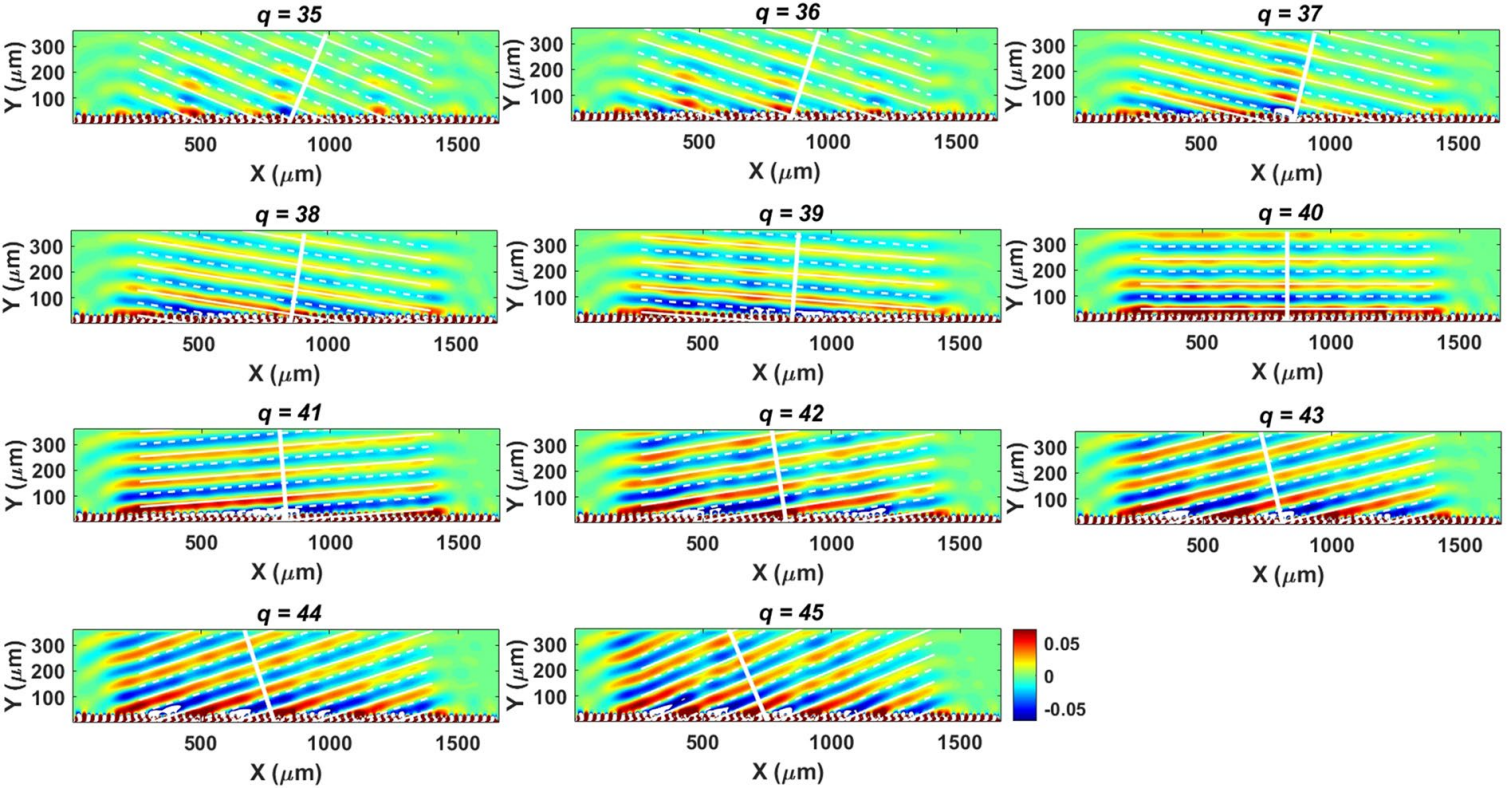
Simulated results for SPE from ESM-excited SPs passing through period gratings. Simulated contours of Fourier transform of Ex fields at 3 THz in x–z plane (cut at the center of gratings in y direction) for different number of gratings ($q$) with electron energy of 29 keV. The thick white lines indicate the predicted directions of emission from Eq. (2).

Subsequently, radiation emitted from ESM-excited SPs passing through period gratings (SPs emission, SPE) is examined (see Fig. 1a). Here the total length of the periodic gratings, *L*, is equal to $2\pi \,r_{g}$. And the period of gratings, G, is equal to ${\text{L}}\,/\,q$, where *q* is the number of gratings. The emission condition can be expressed as^18^

2 $$k_{sp} \; = \;k_{0} \;\sin \theta \; + \;\frac{2\pi }{G}\,\left| {\,n\,} \right|$$

where $k_{0} = \omega /c$ denotes the wavevector of SPE in air and $\theta$ is the emission angle measured from the direction normal to the grating surface ($\theta > 0$ ($\theta < 0$) for forward (backward) emission; *n* denotes the order of emission (the other symbols have been defined above). It has been shown that the frequency and wavevector of excited SPs on the Ge film are 3 THz and $2.0 \times 10^{5}$ rad/m, respectively, for ESM with electron energy of 29 keV. Hence, according to Eq. (2), the SPE will be emitted upward (i.e. $\theta = 0^{0}$) for $q = 40$ ($n = 1$). When $q < 40$ ($q > 40$), the emission will become forward (backward) and $\theta$ increases (decreases) as the value of $q$ decreases (increases). Figure 4 plots the simulated contours of Fourier transform of Ex fields at 3 THz for the values of *q* being between 35 and 45 in x–z plane (cut at the center of gratings in the y direction, see Fig. 1a). In Fig. 4, the thick white lines indicate the predicted directions of emission from Eq. (2). Figure 4 shows that the simulated emission angles of SPE agree with the prediction of Eq. (2) very well.

Next, the SPE of ECM-excited SPs with periodic gratings (Fig. 1b) is examined. As shown above, both the dominant frequency and wavevector of ECM-excited SPs are the same as those of ESM-excited SPs. For the structure of Fig. 1b with $r_{g} = 200$ μm ($p = 40$), it is foreseeable that the SPE will emit upward (along + z direction), forward (along + z direction and $+ \varphi$ direction) and backward (along + z direction and $- \varphi$ direction) for $q = 40$, $q < 40$ and $q > 40$, respectively. The SPE with $\varphi$ dependence implies that the emission possesses OAM. Actually, this emission mechanism is similar to that of the ring resonator using WGM modes^16^. Therefore, the angular phase-matching condition can be applied to predict the quantum number of OAM^16^, i.e.

3 $$\ell = p - q$$

where $\ell$, $p$ and $q$ are the quantum number of OAM, the azimuthal number of optical periods of SPs and the number of gratings, respectively (*p* and *q* have been defined above).

**Figure 5:**
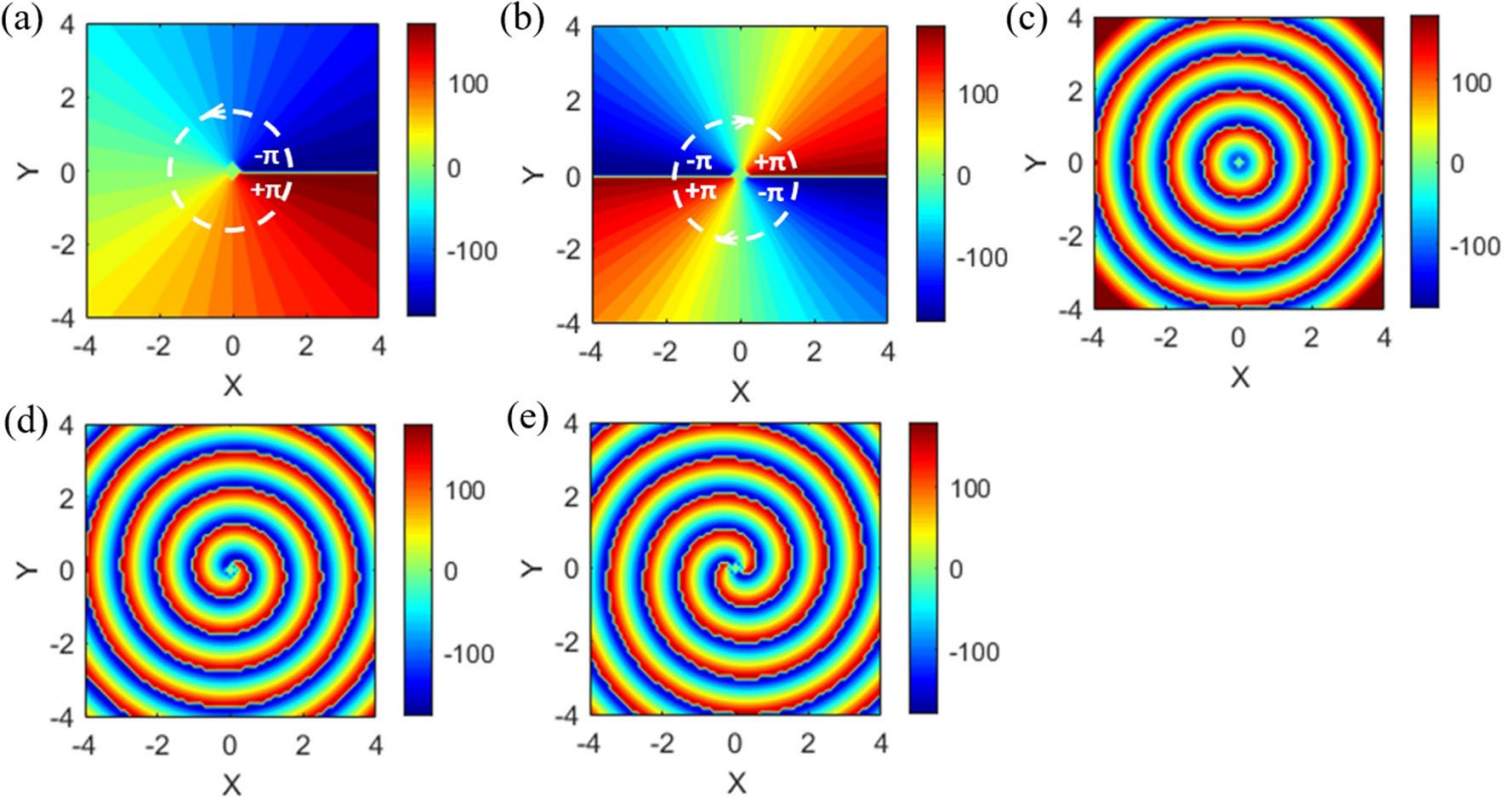
Phase maps for upward emission beams with *φ*-dependent phase distribution on the plane normal to the emission direction. (**a**–**c**) Phase maps of $\ell = + 1$, $\ell = - 2$ and $\ell = 0$ but with pure diffraction effect, respectively. (**d**,**e**) Phase maps of combination of *φ*-dependent phase distribution and pure diffraction effect for $\ell = + 1$ and $\ell = - 2$, respectively.

The quantum number of OAM of emission beam can be identified by examining its phase distribution (phase map). For an upward emission beam (along + z direction) containing OAM, the electric fields on x–y plane has the $\varphi$-dependent phase distribution. Figure 5a and b plot the phase maps of $\ell = + 1$ and $\ell = - 2$, respectively. For $\ell = + 1$ ($\ell = - 2$), the $\varphi ^{\prime}{\text{s}}$ angle increases from $- \pi$ to $+ \pi$ once (twice) in the counterclockwise (clockwise) direction. On the other hand, the pure diffraction effect makes the phase distribution of fields to be a concentric pattern (see Fig. 5c). The combination of $\varphi$-dependent phase distribution of OAM and diffraction effect will cause the phase map to become the one-arm clockwise (two-arm counterclockwise) spiral pattern, Fig. 5d (Fig. 5e), for $\ell = + 1$ ($\ell = - 2$).

**Figure 6:**
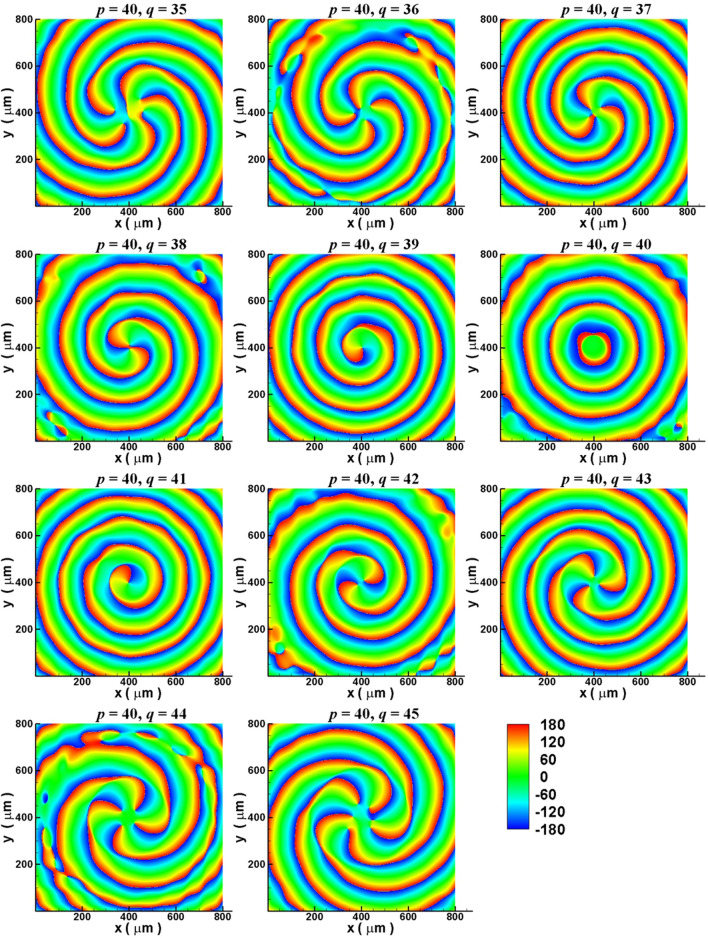
Simulation results for manipulating the quantum number of OAM of ECM-excited SPE by changing the number of gratings. Simulated phase maps of Fourier transformed Ez fields at 3 THz on x–y plane measured at 190 μm above the gratings for the values of *q* varying between 35 and 45 with the electron energy of 29 keV (i.e. $p = 40$, see Table 1).

Subsequently, manipulation of the quantum number ($\ell$) of OAM by changing the number of gratings ($q$) for ECM-excited SPE is investigated [with the electron energy of 29 keV, $r_{g} = 200\;\mu m$, $f_{sp} = 3\;THz$, $k_{SP} = 2.0 \times 10^{5}$ rad/m and $p = 40$ (Table 1)]. Figure 6 presents the simulated phase maps of Fourier transformed Ez fields at 3 THz on x–y plane measured at 190 μm above the gratings (see Fig. 1b) for the values of *q* varying between 35 and 45. Figure 6 shows that, for $q < 40$ ($q > 40$), the phase distribution patterns are clockwise (counterclockwise) spirals and the numbers of arms are equal to the values of $p - q$ ($\left| {p - q} \right|$). These results imply that the SPE containing OAM with the quantum number $\ell = p - q$ [Eq. (3)]. For $p = 40$ and $q = 40$ ($\ell = 0$), the phase map in Fig. 6 displays a concentric phase distribution due to lack of $\varphi$ dependence (i.e. lack of OAM). Figure 6 is consistent with the inference in the previous two paragraphs and demonstrate that the quantum number of OAM can be manipulated by the number of gratings.

**Figure 7:**
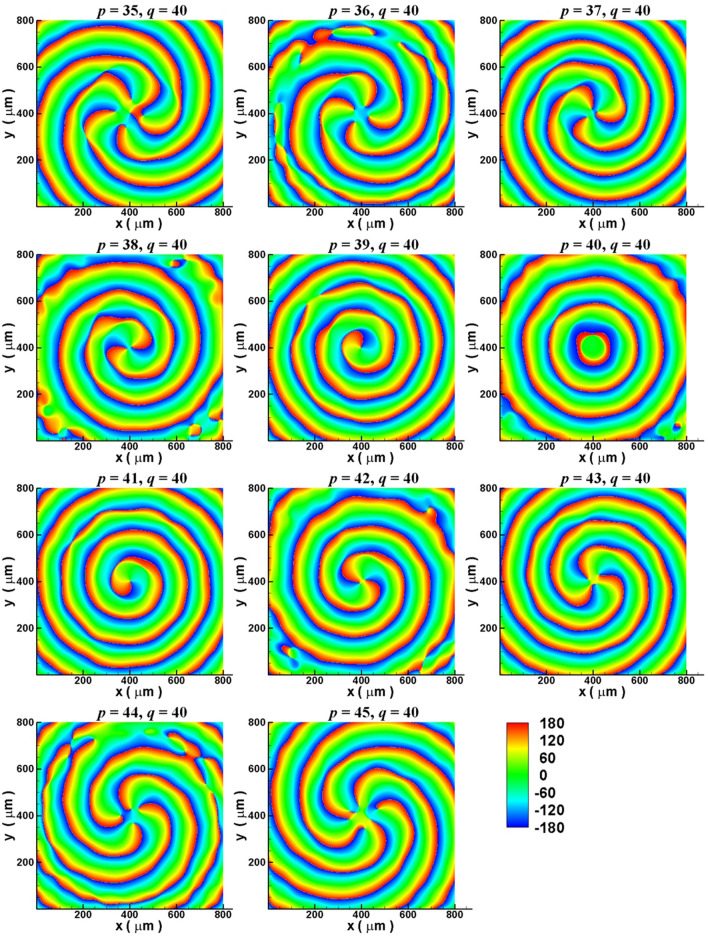
Simulation results for steering the quantum number of OAM of ECM-excited SPE by changing the electron bunch energy. Simulated phase maps of Fourier transformed Ez fields at 3 THz on x–y plane measured at 190 μm above the gratings for the values of *p* being between 35 and 45 (see Table 1) with $q = 40$.

Steering of OAM’s $\ell$ by changing the electron bunch energy (i.e. changing the number of SPs periods along the electron’s orbital, $p$) is also explored. Here $r_{g}$ is also set to $200\;\mu m$ and $q = 40$. The electron energy increases from 22 to 40 keV (the value of $p$ decreases from 45 to 35, see Table 1). These energies make $f_{sp}$ to be within the flat band of dispersion curve of Ge (Fig. 2a, $f_{sp} = 3\;THz$). Figure 7 plots the simulation phase maps of Fourier transformed Ez fields at 3 THz on x–y plane also measured at 190 μm above the gratings for *p* being between 35 and 45. Figure 7 displays that, when $p$’s value increases from 35 to 39, the phase distribution patterns are counterclockwise spirals (i.e. $\ell < 0$) and the number of arms (i.e. the value of $\left| \ell \right|$) decreases from 5 to 1. When $p = 40$, the phase map in Fig. 7 exhibits a concentric phase distribution which demonstrates the diffraction pattern with $\ell = 0$. When $p$’s value further increases from 41 to 45, Fig. 7 shows that the phase distribution patterns become clockwise spirals (i.e. $\ell > 0$) and the number of arms (i.e. the value of $\ell$) increases from 1 to 5. These results also agree with Eq. (3) and suggest that OAM’s quantum number can be steered by electron energy.

The threshold energy for electron bunch to generate SPE with OAM is also estimated. The emission condition of SPE is expressed in Eq. (2). The maximum value of $k_{sp}$ and hence the threshold of electron energy can be determined by $\theta = 90^{o}$. For the typical case in this work (i.e. $q = 40$, $f_{sp} = 3\;THz$ and *n* = 1), the maximum value of $k_{sp}$ is equal to $2.6283 \times 10^{5} \;rad/m$ for $\theta = 90^{o}$. According to Fig. 2a, the threshold energy of electron bunch is 15.259 keV. The range of quantum number of SPE with OAM can also be forecasted by Eqs. (2) and (3). For the typical case of electron energy of 29 keV ($f_{sp} = 3\;THz$, $k_{SP} = 2.0 \times 10^{5}$ and $p = 40$) with ${\text{G}} = 2\pi \;r_{g} /q$ ($r_{g} = 200\;\mu m$) and *n* = 1, The upper (lower) bound of *q*’s value can be obtained by setting $\theta = 90^{o}$ ($\theta = - 90^{\circ}$). The calculated upper and lower bounds of *q*’s values are 52 and 28, respectively. As a result, the quantum number of SPE with OAM (i.e. $\ell = p - q$) is in the range between − 12 and 12.

**Figure 8:**
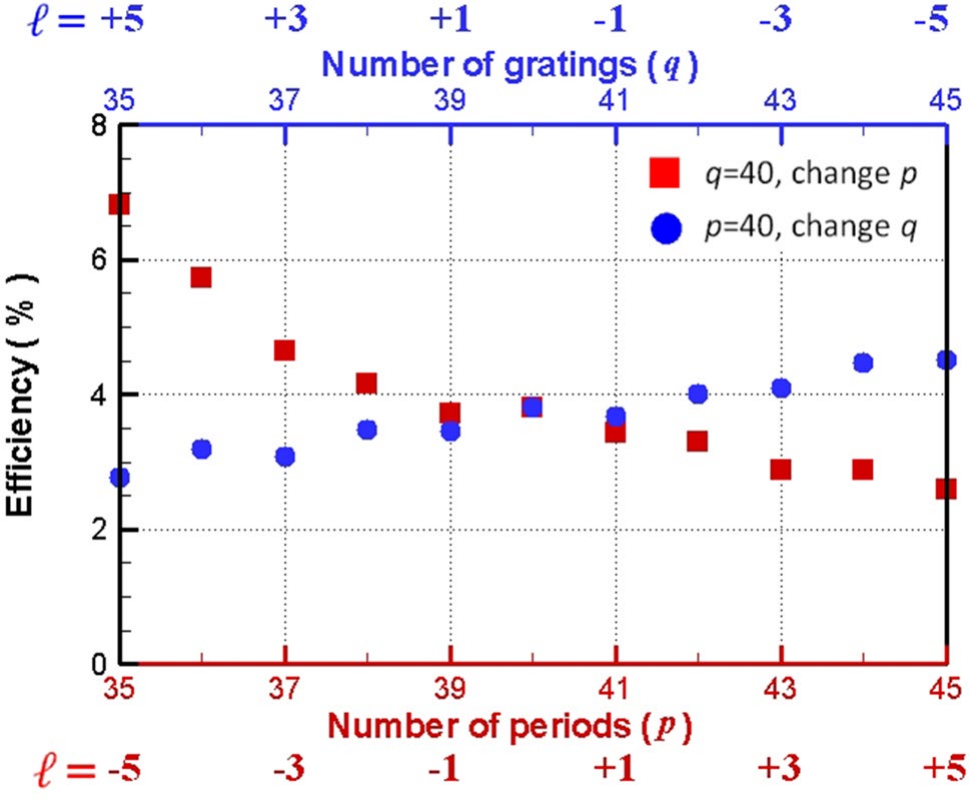
Simulated power conversion efficiencies of ECM-excited SPE. Simulated power conversion efficiencies ($\eta_{p}$) as a function of number of SPs periods ($p$) with $q = 40$ (red square and red bottom x-axis) and as a function of number of gratings ($q$) with $p = 40$ (blue circle and blue upper x-axis).

Finally, the power conversion efficiencies of ECM-excited SPE are investigated. The power conversion efficiency ($\eta_{p}$) can be calculated as^29^

4 $$\eta_{p} = \frac{{P_{OAM} }}{{P_{0} }}$$

where *P*_*OAM*_ is the total Poynting power of SPE integrated over one electron cycle and over the whole surfaces enclosing the upper simulation regions; *P*_*0*_ denotes the total available power of electron bunch in its entire path integrated over one electron cycle (i.e. the same Poynting power except that the structure is removed and measured at 5 μm above the electron bunch). (The distance between the electron bunch and the Ge film is also 5 μm.) The total available power of electron bunch is proportional to its energy loss due to the cyclotron motion in free space. Figure 8 plots the simulated $\eta_{p}$ as a function of number of SPs periods ($p$) with $q = 40$ (red square) and as a function of number of gratings ($q$) with $p = 40$ (blue circle). Figure 8 shows that the values of $\eta_{p}$ are about 3–7% for various simulation cases. For $p = 40$, Fig. 8 displays that $\eta_{p}$ increases with the increase of $q$’ value (the number of gratings). On the contrary, for $q = 40$, Fig. 8 shows that $\eta_{p}$ decreases as the value of $p$ (the number of SPs periods) increases (i.e. $\eta_{p}$ is reduced with the decrease of electron energy). Furthermore, Fig. 8 also reveals that $\eta_{p}$ monotonically increases as the value of $\ell$ ($= p - q$) decreases from + 5 to − 5. These results arise due to that, with respect to a specific number of periods of SPs in the circular orbital, adding more gratings can extract more energy from the SPs and transfer it into radiation. Notably, the extract efficiency of the SPE with OAM, $\eta_{ex}$, can be expressed as $\eta_{ex} = \eta_{p} \times \frac{\Delta E}{{E_{k} }}$, where $\Delta {\text{E}}$ and ${\text{E}}_{k}$ are the energy loss during one cycle and the original energy, respectively, of the electron bunch which performs the cyclotron motion in free space. In the expression of $\eta_{ex}$, the effects of changing electron energy and grating numbers on efficiency have been considered in the term of $\eta_{p}$. The second term in $\eta_{ex}$, $\frac{\Delta E}{{E_{k} }}$, is almost a constant in all of our simulation cases. Therefore, $\eta_{p}$ can reflect the real trend of change of extract efficiency with electron energy and number of gratings. The PIC-FDTD simulation result shows that the electron energy is reduced by around 0.007% during one cycle for ECM exciting SPs. Hence, the upper limit of $\eta_{ex}$ is about 0.007%.

In addition, we also make a comparison between Ref.^16^ and this work to emphasize the innovation of this work. In Ref.^16^, the method to generate the OAM beam is to use a ring resonator which supports the WGMs with adding periodic gratings on the resonator. In this method, a pumping optical beam is incident into the structure and then converted into the radiation with OAM. Each WGM has a specific resonant frequency. Due to this characteristic of WGMs, for a given number of gratings in the ring resonator, the injected frequency must change with the quantum number of OAM. Conversely, in this work, the OAM beam is generated by using an electron bunch to excite SPs on a Ge film and subsequent SPE from periodic Ge gratings. The number of periods of SPs in the circular orbit (similar to the role played by WGMs) can be manipulated by electron’s energy and cyclotron frequency. More importantly, by using the flat band of dispersion relation of SPs, for a giving number of gratings, a continuous change of quantum number of OAM with a designed frequency can be achieved by only changing the electron energy. It is also noted that, propagation of spoof surface plasmons (SSPs) on ring or straight waveguides with sub-wavelength structures such as metallic rods and slits have been investigated in the literature^30–33^. In the operation frequency of SSPs, the metals behave like perfectly electric conductors (PECs). The dispersion relations of SSPs strongly depend on the depth and period of the sub-wavelength structures. However, in this work, SPs on the Ge film are real SPs. Their dispersion relations have been given in Eq. (1). Furthermore, the electron bunch moves under the Ge film. It cannot directly excite any SSPs mode on the periodic Ge gratings, even if it really exists. Therefore, it doesn’t need to consider the SSPs in this work. Actually, the simulation properties of SPs and SPE ($k_{sp}$, $f_{sp}$ and $\theta$) agree with those predicted by Eqs. (1) and (2). The observed phenomena have been clearly explained by Eqs. (1)–(3).

## Discussion

ECM-excited SPs on a Ge thin film in THz region and subsequent SPE by adding Ge gratings on the film are explored by FDTD and PIC-FDTD simulations in this work. The optical properties of ECM-excited SPs are the same as those of ESM-excited SPs. For operating at the flat band of SPs’ dispersion curve of the Ge film, changing the electron energy will only change $k_{sp}$ of SPs and hence the number of periods of SPs on the circular orbital. When the periodic gratings are deposited on the Ge film along the electron’s circular orbital, the emitted SPE possesses the OAM. The number of arms and chirality of the spiral patterns in phase map (i.e. the quantum number of OAM) are determined by the difference between the number of SPs’ periods and the number of gratings. Manipulations of the quantum number of OAM by changing the number of gratings for a fixed electron energy and by changing the electron energy for a fixed number of gratings are also demonstrated. This work provides an active OAM source and it is not required to launch circularly polarized beams or pumping beams into the structure.

## Methods and materials

The FDTD program MEEP is used in this study^26^. The simulations are performed in Cartesian x–y–z coordinate system with the grid sizes in all directions to be set as 1 μm. The perfectly matched layers (PMLs) are applied to enclose the whole simulation region. The optical property of Ge is described by Drude model with the following form:

5 $$\varepsilon {{(\omega }})\; = \;\varepsilon_{\infty } \;(1.0 - \frac{{\omega_{p}^{2} }}{\omega \,(\omega + i\gamma )})$$

In Eq. (5), the high-frequency dielectric constant ($\varepsilon_{\infty }$), plasma frequency ($\omega_{p}$) and collision frequency ($\gamma$) are set as 16.55, $2.1627 \times 10^{13}$ rad/s and $2.746 \times 10^{11}$ rad/s, respectively^28^. In the simulation, the electron bunch is modeled by a dipole source which moves along a circular orbit with $r_{g} = 200$ μm and predefined $\omega_{c}$ (i.e. using “change-sources!” function in MEEP). To maintain $r_{g} = 200$ μm, the values of ${\text{B}}_{{0}}$ vary with the electron energy and are within the range of 2–4 T (see Table 1). However, with these values of ${\text{B}}_{{0}}$, the cyclotron frequency of electrons in the Ge film is much smaller than their plasma frequency. (The effective mass of electron in Ge, $m_{Ge}$, is equal to $1.64 \times {\text{m}}_{{0}}$, where $m_{0}$ is the mass of electron in vacuum^34^. The cyclotron frequency of electron in Ge with the external magnetic field of 2.8 T is $3.0 \times 10^{11} \;rad/s$. And the plasma frequency of electron in Ge is $2.163 \times 10^{13} \;rad/s$.) Therefore, the magnetized effect of electrons in the Ge film is neglected. The optical property of Ge can be described by the Drude model.

The PIC-FDTD program VORPAL is also adopted in this work to model the interaction of electrons with the electromagnetic fields of SPs and the applied external magnetic fields^35^. The ECM will be automatically generated when the electron has an initial velocity and an external magnetic field being applied perpendicular to the direction of electron velocity. In PIC-FDTD simulation, the grid sizes in x and y directions and in z direction are set as 2 μm and 1 μm, respectively. The other geometry and material settings in VORPAL are the same as those in MEEP^36^. At the beginning, a total of 100 macro-particles (each macro-particle represents 10 real electrons) are uniformly distributed in a $4\;\mu m \times 4\;\mu m \times 2\;\mu m$ volume. In the subsequent process, the macro-particles perform the cyclotron motion with $r_{g} = 200$ μm and excite the SPs along their orbital (see the movie in Supplementary Information). In the PIC-FDTD simulation, the space charge effect of the macro-particles is included in the calculation. However, in our simulation case, the space charge effect of 100 macro-particles with 10 real electrons in each macro-particle is weak so that it doesn’t affect the motion of macro-particles. The optical properties of SPs on a Ge film obtained from VORPAL simulation agree with those from MEEP simulation. Furthermore, the reduction of macro-particles’ energy during one cycle can be calculated under VORPAL simulation.

## Supplementary information

Supplementary Legend.

Supplementary Movie.
