# Supplementary material for: Electron cyclotron motion excited surface plasmon and radiation with orbital angular momentum on a semiconductor thin film
Source: Sci Rep. 2020 Oct 7;10:16768. doi: 10.1038/s41598-020-73725-6 (PMC7541642; doi:10.1038/s41598-020-73725-6)
Supplement: Supplementary file 1 — Supplementary Legend. [file 41598_2020_73725_MOESM1_ESM.doc]

Supplementary Information

**Electron cyclotron motion excited surface plasmon and radiation with orbital angular momentum on a semiconductor thin film**

*Yung-Chiang Lan, Chia-Hui Shen, and Chih-Min Chen*

**1. Movie S1**

**S1.** Movie of electron bunch to perform cyclotron motion under a Ge film and its excitation of SPs on the film (by observing the Ez field on top surface) from PIC-FDTD simulation (29-keV electron energy and $r_{g}=200 \mu m$)
